# Perinatal Hemolytic Disease Due to Anti-Jkb: A Case Report

**DOI:** 10.7759/cureus.83787

**Published:** 2025-05-09

**Authors:** Flavia M Bandeira, Camila A Mesquita, Kallic B Fonseca, Helena P Magalhães, Sandro Artur F Silva, Fernando S Silva

**Affiliations:** 1 Hematology and Transfusion Medicine, Faculty of Medical Sciences, Rio de Janeiro State University, Rio de Janeiro, BRA; 2 Transfusion Medicine, Pedro Ernesto University Hospital, Rio de Janeiro State University, Rio de Janeiro, BRA; 3 Medical School, Faculty of Medical Sciences, Rio de Janeiro State University, Rio de Janeiro, BRA; 4 Immunohematology, Pedro Ernesto University Hospital, Rio de Janeiro State University, Rio de Janeiro, BRA

**Keywords:** erythrocyte phenotyping, immunohematology, maternal alloimmunization, neonatal jaundice, perinatal hemolytic disease, phenotyped red blood cells

## Abstract

This is a case report of a pregnant woman, with a history of cesarean section in a previous twin pregnancy, admitted due to premature rupture of membranes and investigation of anemia during her fifth pregnancy. During the cesarean section, she experienced significant hemorrhage, prompting a request for a red blood cell concentrate. During pre-transfusion testing, it was found that she was O positive, and an anti-erythrocyte antibody was identified, specifically anti-Jkb. It is noteworthy that despite her numerous previous pregnancies, there was no record of indirect antiglobulin testing (IAT) or irregular antibody screening during prenatal care. The outcome for twin 1 was perinatal asphyxia, resulting in death within 24 hours. Twin 2 developed jaundice and anemia, requiring phototherapy and red blood cell transfusions. This case underscores the need to consider clinical history and the importance of performing IAT in pregnant women, regardless of blood type.

## Introduction

Perinatal hemolytic disease (PHD), also known as hemolytic disease of the newborn (HDN), is a condition characterized by the destruction of fetal or neonatal red blood cells due to maternal antibodies that cross the placenta. This condition most frequently arises from Rh or ABO blood group incompatibilities, although other blood groups may also be involved. This pathological process can result in significant clinical manifestations, including jaundice, anemia, and, in severe cases, hydrops fetalis, necessitating prompt diagnosis and intervention. Maternal-fetal erythrocyte alloimmunization is well known among neonatologists, pediatricians, and obstetricians. Related to PHD, the absence of the maternal D antigen was described in the 1940s due to its consequence in a pregnant woman with an RhD-positive fetus and the catastrophic hemolysis it could cause, leading to fetal death in a condition called fetal erythroblastosis [1]. Despite various preventive measures for PHD due to anti-D, such as identifying anti-D in RhD-negative pregnant women during prenatal care, assessing fetal circulation through transcranial Doppler, intrauterine transfusion, or exchange transfusion (EXT), and administering anti-D immunoglobulin to the non-alloimmunized mother post-delivery, severe cases still occur [2,3].

Almost 400 erythrocyte surface antigens associated with nearly 50 blood group systems have been described to date [4]. Several of these antigens are associated with PHD and can severely affect the fetus and newborn. Maternal exposure to antigens present in the newborn's red blood cells but absent in maternal red blood cells can lead to the formation of anti-erythrocyte antibodies, usually IgG. Consequently, these antibodies cross the placental barrier, potentially leading to the destruction of fetal erythrocytes or even blocking the production of erythroblasts in the fetal bone marrow [5]. This immune response is individual and not always predictable. In addition to this form of alloimmunization, women can also become sensitized through transfusions, during pregnancy, and, more rarely, through natural antibody formation, which is uncommon but possible [6].

A systematic review [7] showed that the anti-D alloantibody (40%) was the most prevalent, followed by anti-A (32%), anti-B (11%), anti-K (7.72%), anti-c (2.30%), anti-E (1.40%), anti-C (1%), anti-M (0.40%), anti-G (0.20%), anti-Dib (0.16%), anti-Jka and anti-U (0.10%), anti-e, anti-At, anti-Jk3, anti-Wra, anti-Fya, anti-Ku, anti-Rd, anti-Jkb, anti-Vel, anti-Cw, anti-KEAL, and anti-SARA, all at 0.05% each, as well as associations of anti-D+anti-C (0.70%), anti-c+anti-E (1.20%), and anti-D+anti-E (0.16%). Also observed were anti-D+anti-Lea, anti-C+anti-G, anti-K+anti-Jka, anti-L+anti-Lea, and anti-K+anti-Leb, each at 0.05%. In routine prenatal care, the presence of irregular antibody (PAI) is generally screened through the indirect antiglobulin test (IAT) in RhD-negative pregnant women; however, it is advisable to perform screening in high-risk pregnancies [7]. This case report aims to highlight the importance of conducting PAI testing in prenatal care even for RhD-positive pregnant women to enable intervention for the prevention of severe PHD, fetal death, anemia, and neonatal jaundice due to maternal-fetal incompatibility. Such a recommendation should be considered for high-risk pregnancies; however, a cost-benefit analysis is warranted before implementation. Furthermore, it emphasizes that transfusion planning for the mother, fetus, and newborn can be carried out with the necessary care and criteria. The objective is to report a case of maternal alloimmunization due to anti-Jkb, transmitted to the fetuses in a twin pregnancy, leading to PHD.

## Case presentation

This case describes a PHD due to maternal alloimmunization by anti-Jkb. The obstetric history included a previous twin pregnancy and a miscarriage. In the pregnancy that led to the description of this case, she did not attend prenatal care; however, the patient developed moderate anemia, which was investigated in an outpatient setting. At about six weeks before delivery, she presented with premature rupture of membranes, leading to her admission for clinical monitoring and fetal assessment in the obstetric emergency room. The patient developed hypertension and chorioamnionitis, which led to the interruption of the pregnancies at 30 weeks. She gave birth to two female twin newborns classified as moderately preterm (born between 31 and 36 weeks). The birth weight of twin 1 was 1180 g, APGAR 0/4/6, whereas twin 2 weighed 1330 g, APGAR 1/5/7. Both newborns presented with perinatal asphyxia, and immediately postpartum, the mother experienced significant hemorrhage (Hb 6 g/dL), prompting a request for red blood cell concentrate transfusions for her and for the second twin (Hb 7 g/dL), who was born with severe anemia in addition to signs of perinatal asphyxia. During pre-transfusion testing, it was observed that the mother and both twins were O positive, with both twins having positive direct antiglobulin tests (DAT) (DG Gel Coombs and Saline DG Gel Sol by Grifols Diagnostic Solutions (Emeryville, California, United States)) and the presence of anti-Jkb in the hemagglutination panel, conducted with eluate serum (DiaCidel by Bio-Rad (Hercules, California, United States)). The mother tested positive for irregular antibodies through the IAT (Serascan Diana 2 by Grifols Diagnostic Solutions), with the identification of anti-Jkb (Identisera Diana and Identisera Diana P by Grifols Diagnostic Solutions). Anti-Jkb titration was not performed. The mother denied any complications in previous pregnancies or transfusion history, stating that children born from previous pregnancies had no complications of anemia or jaundice. The outcome for twin 1 was perinatal asphyxia, resulting in death within 24 hours. Twin 2 developed jaundice and anemia, requiring phototherapy and red blood cell transfusions until discharge from the neonatal intensive care unit (ICU). As the pregnant woman did not attend prenatal care, there was no record of fetal condition. During the admission to the obstetric emergency room, a request for an IAT was made; however, the result was not checked. A positive IAT was evidenced with the identification of anti-Jkb only during the maternal post-partum transfusion need (Hb 6 g/dL).

## Discussion

The case presented is noteworthy as it involves an RhD-positive pregnant woman with a history of multiple pregnancies, who was not evaluated during prenatal care for the PAI. She gave birth to twin newborns, both developing PHD due to anti-Jkb. At delivery, twin 1 weighed 1180 g and twin 2 weighed 1330 g. Twin 1 died within the first 24 hours with severe anemia and perinatal asphyxia, and twin 2 was discharged after several transfusions and intensive care support due to anemia, perinatal asphyxia, and infection. Although PHD due to Jkb is typically mild to moderate, this case presented with unusually severe manifestations.

With the introduction of anti-RhD immunoglobulin for RhD-negative women, there has been a reduction in cases of PHD due to anti-D, which continues to be the prototype for this type of condition involving blood incompatibility between the mother and the fetus. A study conducted in Canada highlights that maternal alloimmunization due to anti-D [5,6] remains highly relevant [2]. A retrospective study in northern India identified that 22.7% of pregnant women with anti-D also presented with other alloantibodies for the Rh system, with the most common being anti-C, anti-E, and anti-c [8]. The emergence of PHD cases due to other erythrocyte antigens has been reported in the literature and deserves attention from obstetricians and neonatologists regarding the identification of alloimmunized pregnant women and the risk of PHD [9,10]. The variation in erythrocyte phenotypes is related to ethnic profiles. A study conducted in Nigeria showed variable distribution in the frequency of Kidd system antigens among pregnant women of various ethnicities, even when compared to Caucasian and Asian populations [11]. The performance of erythrocyte phenotyping in pregnant women and the screening for irregular antibodies, even in RhD-positive pregnant women, has been recommended by various authors, since PHD can occur severely in women alloimmunized by other erythrocyte antigens [11,12].

Discovered in 1951, the Kidd system has two opposing antigens, Jka and Jkb, and also a third frequent antigen, Jk3, which is expressed in all individuals except in the rare cases of the null Kidd phenotype. The function of the glycoprotein that carries the Kidd system antigens is to transport urea in the endothelial cells of the renal vasa recta as well as in erythrocytes [13]. The three Kidd antigens can cause PHD, but they are generally described as causing mild cases [12-14]. Some case reports mention the occurrence of PHD due to anti-Jkb evolving moderately, although reports of severe cases also exist [15-17]. In pregnant women who maintain antibody titers up to 1:8 or 4 IU/ml, the fetus is considered at minimal risk of severe antenatal involvement by PHD [18].

In the context of prenatal care, several critical considerations must be emphasized to optimize maternal and fetal outcomes. The absence of prenatal visits in this case resulted in a lack of documentation regarding fetal status, which is paramount for the early identification of potential complications such as intrauterine growth restriction, congenital anomalies, or placental insufficiency. Regular prenatal assessments facilitate the monitoring of both maternal and fetal health parameters, enabling timely interventions that can significantly mitigate risks. Furthermore, had the alloimmunization been identified earlier upon the request for an IAT, appropriate management strategies could have been initiated. This includes the implementation of non-invasive fetal monitoring methods such as transcranial Doppler ultrasound to evaluate fetal status and determine the risk of conditions like fetal anemia, which can arise from maternal alloimmunization. The oversight regarding the follow-up of the IAT results, only being addressed during the postpartum period when the mother exhibited severe anemia (hemoglobin 6 g/dL), highlights a critical gap in the standard of care. The detection of anti-Jkb at this late stage complicates maternal management and poses significant risks to future pregnancies due to the potential for alloimmunization. The importance of structured prenatal care cannot be overstated; it is integral for the early detection and management of maternal-fetal risks, thereby promoting favorable health outcomes. Comprehensive prenatal care protocols should include routine laboratory evaluations, meticulous follow-up on test results, and effective communication among healthcare providers to ensure the timely identification and management of conditions such as alloimmunization. In Brazil, the miscegenation of the population is a factor to be considered, including in public health policy decisions. The case described demonstrates the relevance and necessity of considering both clinical and obstetric history, as well as racial factors, in implementing prenatal care and tests that can mitigate the risk of PHD in the fetus and newborn.

## Conclusions

The presented case reinforces the need for a more detailed assessment regarding the risk of maternal alloimmunization and the subsequent potential development of PHD. Previous transfusion history and prior pregnancies must be taken into account during prenatal care, as well as the development of obstetric protocols aimed at identifying the potential risk of PHD even in non-RhD-negative pregnant women. Access to prenatal care, particularly for high-risk pregnancies, should be ensured.
